# Blood Urea Nitrogen Is Associated with In-Hospital Mortality in Critically Ill Patients with Acute Exacerbation of Chronic Obstructive Pulmonary Disease: A Propensity Score Matching Analysis

**DOI:** 10.3390/jcm11226709

**Published:** 2022-11-13

**Authors:** Mohan Giri, Lin He, Tianyang Hu, Anju Puri, Xiaozhuo Zheng, Haiyun Dai, Shuliang Guo

**Affiliations:** 1 Department of Respiratory and Critical Care Medicine, The First Affiliated Hospital of Chongqing Medical University, No 1 Youyi Road, Yuzhong, Chongqing 400016, China; 2 Department of Respiratory and Critical Care Medicine, Chongqing University Fuling Hospital, Chongqing 408000, China; 3 Precision Medicine Center, The Second Affiliated Hospital, Chongqing Medical University, Chongqing 400010, China

**Keywords:** acute exacerbation of chronic obstructive pulmonary disease, blood urea nitrogen, critical care, intensive care unit, mortality, propensity score matching

## Abstract

Background: Elevated blood urea nitrogen (BUN) level is associated with a higher risk of mortality in various diseases; however, the association between BUN level and in-hospital mortality in patients with acute exacerbation of chronic obstructive pulmonary disease (AECOPD) admitted to the intensive care unit (ICU) is not known. This study aimed to investigate the relationship between BUN level and in-hospital mortality in patients with AECOPD admitted to the ICU. Methods: In this retrospective cohort study, AECOPD patients were identified from the Medical Information Mart for Intensive Care (MIMIC-IV) database. Multivariate regression was used to elucidate the relationship between BUN level and in-hospital mortality, and propensity score matching (PSM) was used to adjust confounders. Receiver operating characteristics and Kaplan–Meier curves were used to evaluate the relationship between BUN level and in-hospital mortality. Results: Data from 1201 patients were analyzed. The all-cause in-hospital mortality was 13.7%. BUN levels were significantly higher in non-survivors compared to the survival group before (*p* < 0.001) and after (*p* = 0.005) PSM. Multivariate analysis indicated that elevated BUN levels were independently associated with increased risk of in-hospital mortality both before (*p* = 0.002) and after (*p* = 0.015) PSM. The optimal BUN cut-off value for in-hospital mortality in critical patients with AECOPD before (>23 mg/dL) and after (>22 mg/dL) PSM was comparable. Compared with the low BUN group, the hazard ratio (HR) of the high BUN group was 1.8987 (before PSM) and 1.7358 (after PSM). Conclusions: Higher BUN levels were significantly associated with an increased risk of in-hospital mortality in critically ill patients with AECOPD. As a widely available and rapidly measured biomarker, BUN may be useful in the risk stratification of critically ill AECOPD patients. The results need to be verified in prospective studies.

## 1. Introduction

Chronic obstructive pulmonary disease (COPD) is a heterogeneous disease characterized by persistent respiratory symptoms and progressive airflow limitation, with episodes of exacerbations leading to substantial morbidity and mortality [1,2]. With an estimated 3.2 million deaths annually, COPD is the third leading cause of death worldwide [3]. Patients with acute exacerbations of COPD (AECOPD) often require unscheduled admission to the hospital or intensive care unit, incurring a substantial economic burden on individuals and society. The in-hospital mortality rate for AECOPD patients varies between 4% and 30% [4,5,6]. Previous studies reported various prognostic factors associated with in-hospital mortality of patients with AECOPD, including age, comorbidities, prior hospitalization due to AECOPD, exacerbation severity, and several laboratory parameters [7,8]. However, less invasive and technically easier laboratory parameters to predict in-hospital mortality, especially in the intensive care unit, represent an unmet need to minimize the negative impact of AECOPD.

Blood urea nitrogen (BUN) is a nitrogenous terminal product of protein metabolism formed in the liver and excreted by the kidneys. In addition to estimating renal function, BUN is an efficient indicator of neurohormonal activity [9]. Impaired cardiorenal function and neurohormonal activation may result in a high BUN level, which has been linked to mortality in various diseases [9,10]. Several previous studies suggested that BUN is a prognostic indicator for mortality in multiple disorders, including heart failure, aortic dissection, pancreatitis, gastrointestinal bleeding, diabetes mellitus, etc. [11,12,13,14]. Furthermore, recent studies have revealed that BUN is a significant predictor of mortality and disease severity in critically ill patients admitted with respiratory disorders such as coronavirus disease 2019 (COVID-19), hospital-acquired pneumonia, and community-acquired pneumonia (CAP) [15,16]. A recent prospective study demonstrated that the CURB-65 score is a valuable and simple tool for predicting 30- and 90-day mortality in COPD exacerbation [17]. BUN is one of the most crucial parameters in the CURB-65 score. Several factors, including hypoxia, chemoreflex, hypercapnia, and systemic inflammation, may impair cardiorenal function and neurohumoral regulation in patients with COPD exacerbations, resulting in a high BUN level [9,18].

Despite the above evidence, to our knowledge, no study has examined the association between BUN level and mortality in critically ill AECOPD patients. Therefore, we hypothesized that higher BUN levels are associated with an increased risk of in-hospital mortality in intensive care patients with AECOPD. We used the Medical Information Mart for Intensive Care—IV (MIMIC-IV) database [19] to investigate the relationship between BUN levels and in-hospital mortality in patients with AECOPD admitted to the intensive care unit (ICU).

## 2. Methods

### 2.1. Data Source

This retrospective cohort study was based on the MIMIC-IV database (version 1.0), which contains data from patients admitted to the intensive care unit of Beth Israel Deaconess Medical Center in Boston, Massachusetts, between 2008 and 2019 [19]. One author (MG) completed the training course “protecting human research participants” to access the database and was responsible for data extraction (certification number: 45355193). The establishment of the MIMIC-IV database has been approved by the institutional review boards of both Beth Israel Deaconess Medical Center and Massachusetts Institute of Technology Affiliates. Informed consent was waived because all the patients in the database are anonymous.

### 2.2. Study Population and Data Extraction

We included all adult patients (≥18 years of age) diagnosed with acute exacerbation of chronic obstructive pulmonary disease (AECOPD) at hospital admission based on the International Classification of Diseases version 9 and 10 diagnosis codes (49121, J440, J441) in the MIMIC-IV database. The exclusion criteria were (i) patients with repeated ICU admissions, (ii) length of ICU stay <24 h, and (iii) more than 10% of individual data missing.

Navicat Premium (version 15.0) was used to extract the raw data of patients diagnosed with AECOPD from the MIMIC-IV database (version 1.0). The extracted data included demographics, vital signs, laboratory tests, comorbidities, and intervention. The following demographic data were extracted: age, gender, length of hospital stay, length of ICU stay, and hospital death sign. Monitoring parameters such as heart rate, blood pressure, respiratory rate, and oxyhemoglobin saturation (SpO2) were also collected. The extracted laboratory data include routine blood tests, glucose, creatinine, blood urea nitrogen, and bicarbonate. Comorbidities including hypertension, diabetes, congestive heart failure, coronary artery disease, chronic kidney disease, severe liver disease, obesity, malignant cancer, and cerebrovascular disease were extracted. Information on whether the patients were on mechanical ventilation was also extracted. We used the average value for the variable that was assessed multiple times on the first day of admission.

### 2.3. Statistical Analysis

The Kolmogorov–Smirnov test was used to determine if variables were normally distributed. If the data were normally distributed, we reported it as a mean ± standard deviation (SD) and used the independent sample t-test to compare all variables. When the data were not normally distributed, the variables were expressed as the median with interquartile range (IQR), and the Mann–Whitney test was used. Categorical variables were presented as total numbers and percentages. The baseline characteristics of the survival group and death group were evaluated by the chi-square test or Fisher’s exact test for categorical variables and the Mann–Whitney test for continuous variables. We carried out a binomial logistic regression analysis to determine the impact of BUN on in-hospital mortality in patients with AECOPD. Parameters with *p* < 0.1 in the univariate analysis and potential confounders determined by clinical expertise were included in multivariable logistic regression. Propensity score matching (PSM) was used to adjust for confounders between the survival and death groups. Confounders such as age, gender, hemoglobin, white blood cells, platelets, creatinine, red cell distribution width, hematocrit, bicarbonate, hypertension, diabetes, congestive heart failure, coronary artery disease, chronic kidney disease, severe liver disease, obesity, malignant cancer, cerebrovascular disease, heart rate, mean arterial pressure, the respiratory rate on the first day of admission, and mechanical ventilation were included in PSM. We used 1:1 nearest-neighbor matching without replacement within a caliper of 0.05 SD of the logit of the propensity score to create matched cohort. Logistic regression analysis was also performed on the matched cohort. Receiver operating characteristic (ROC) curves were generated to evaluate the association of BUN level with in-hospital mortality, and the area under the curve (AUC) was calculated. The optimal cut-off values of BUN associated with longer hospital mortality were determined by the Youden index. The BUN cut-off value was used to divide all patients into two groups: the high BUN group and the low BUN group. Survival analysis was performed using the Kaplan–Meier method, and the log-rank test was used to compare survival rates between high BUN and low BUN groups. Statistical analyses were performed with SPSS version 26.0 software and MedCalc version 19.6. A *p*-value of <0.05 was considered significant.

## 3. Results

### 3.1. Baseline Characteristics

**Figure 1:**
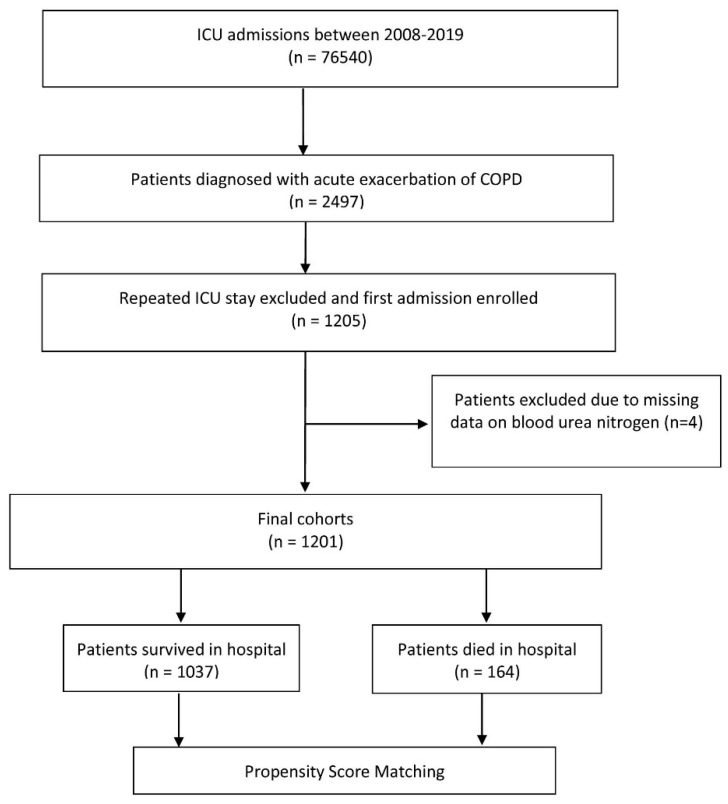
Flow chart of the patient selection process.

Among 76,540 records of ICU admissions in the MIMIC-IV database, we included a total of 1201 critically patients with AECOPD in the present study. The flowchart of the patient selection is presented in Figure 1. The median age of the patients was 72 years (range 41 to 97), and 50.2% were male. The overall rate of in-hospital mortality was 13.7% (164/1201). Table 1 summarizes the characteristics of the survival and death groups. No statistically significant differences were observed (all *p* > 0.05) between the survival and death groups regarding gender and the length of hospital stay. Patients in the death group had higher age, creatinine, red cell distribution width, and heart rate than those in the survival group (all *p* < 0.05) and lower mean arterial pressure, hemoglobin, platelets, hematocrit, and bicarbonate levels (all *p* < 0.05). Compared with the survival group, the death group had a significantly higher prevalence of prior cancer (26.8% vs. 12.7%, *p* = 0.001) and more often required mechanical ventilation during their hospital stay (40.2% vs. 31.5%, *p* = 0.031). Patients in the death group had significantly higher baseline BUN levels than those in the survival group.

### 3.2. Propensity Score Matching Analysis

Propensity score matching was used to balance the baseline characteristics, and 159 patients were allocated to each group. After PSM, most of the baseline characteristics were comparable between the death and survival groups (all *p* > 0.05). However, the length of ICU stay was longer in the death group than in the survival group even after the PSM (*p* < 0.001). Similarly, BUN levels remained significantly higher in the death group compared to the survival group (*p* < 0.05). The baseline characteristics are shown in Table 1.

### 3.3. Logistic Regression Analysis

The univariable and multivariable relationships between patient characteristics and in-hospital mortality before PSM are shown in Table 2. Prior to matching, BUN was a risk factor for in-hospital mortality in ICU patients with AECOPD in univariable analyses (OR:1.018, 95% CI:1.010–1.025, *p* < 0.001), and this risk persisted in multivariable analyses (OR:1.014, 95% CI:1.005–1.022, *p* = 0.002). After PSM, BUN was still a risk factor for in-hospital mortality in patients with AECOPD in univariable analyses (OR:1.016, 95% CI:1.004–1.027, *p* = 0.008) and multivariable analyses (OR:1.015, 95% CI:1.003–1.027, *p* < 0.05). Table 3 presents relevant factors associated with in-hospital mortality in patients with AECOPD after PSM.

### 3.4. Receiver Operating Characteristic Analysis

**Figure 2:**
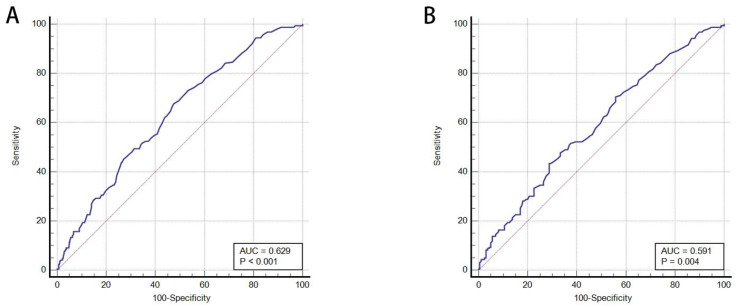
(**A**) Receiver operating characteristic (ROC) curve for BUN (before propensity score matching); (**B**) ROC curve for BUN (after propensity score matching). AUC, area under the curve.

A receiver operating characteristic (ROC) curve was drawn, and the area under the curve was calculated to assess the relationship between BUN level and in-hospital mortality. Before PSM, the optimal cut-off value of BUN level for in-hospital mortality was 23 mg/dL with a sensitivity of 67.68% and specificity of 52.53%. The AUC was 0.629 (95% CI: 0.601–0.656, *p* < 0.001) (Figure 2). After PSM, the AUC value was 0.591 (95% CI: 0.535–0.646; *p* = 0.004), with sensitivity and specificity of 70.44% and 44.03%, respectively, and an optimal BUN cut-off value of 22 mg/dL (Figure 2).

### 3.5. Survival Analysis

**Figure 3:**
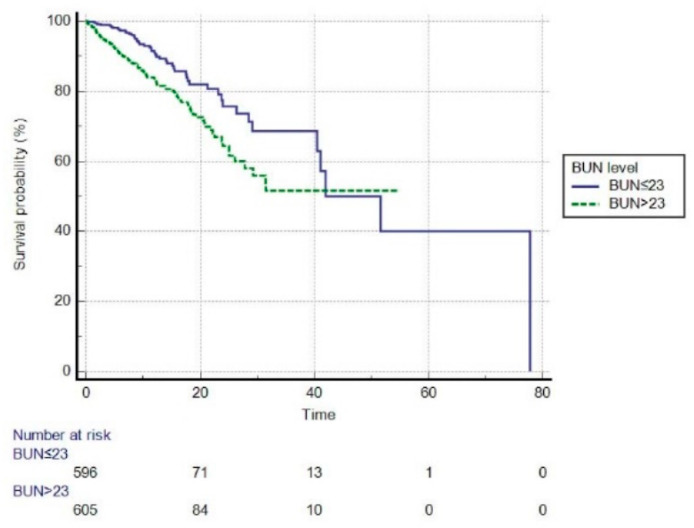
The Kaplan–Meier survival curve of high and low BUN groups (before propensity score matching, log-rank *p* < 0.001). BUN, blood urea nitrogen (mg/dL).

**Figure 4:**
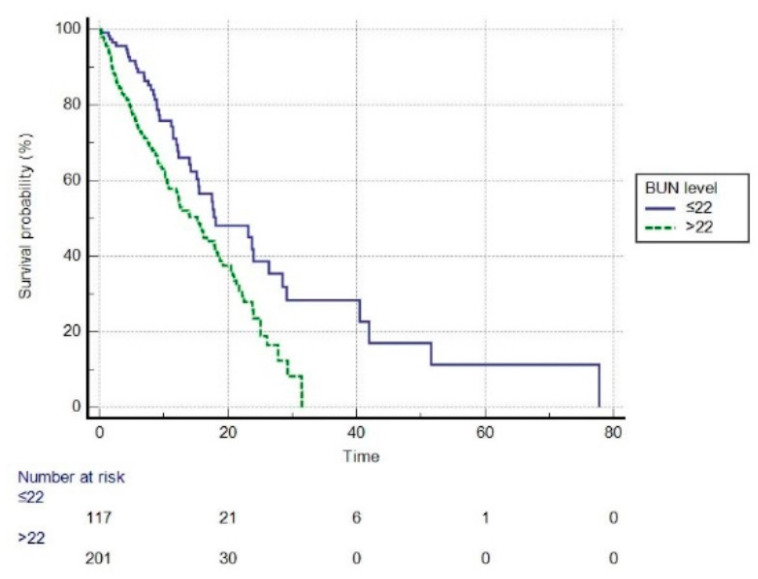
The Kaplan–Meier survival curve of high and low BUN groups (after propensity score matching, log-rank *p* < 0.001). BUN, blood urea nitrogen (mg/dL).

Before PSM, based on the optimal cut-off value of 23 mg/dL, patients were divided into high BUN (BUN level > 23 mg/dL) and low BUN (BUN level ≤ 23 mg/dL) groups. We performed a survival analysis using Kaplan–Meier plots in the high and low BUN groups (Figure 3). The mean survival time was 36.418 days (95% CI: 33.120–39.71) in the high BUN group vs. 49.522 days (95% CI: 40.919–58.126) in the low BUN group, and statistically significant differences were found between the two groups (log-rank test, *p* < 0.001). Compared to the low BUN group, the high BUN group had a hazard ratio (HR) of 1.8987 (95% CI: 1.3975–2.5796, *p* < 0.01). After PSM, the optimal cut-off value for BUN was 22 mg/dL (Figure 4). The high BUN group had a mean survival time of 15.183 days (95% CI: 13.550–16.816), whereas the low BUN group had a mean survival time of 27.087 days (95% CI: 19.926–34.249), and the difference was statistically significant (log-rank test, *p* < 0.001). Compared with the low BUN group, the HR of the high BUN group was 1.7358 (95% CI: 1.2671–2.3780, *p* < 0.01).

## 4. Discussion

To the best of our knowledge, this is the first study to investigate the associations between BUN and in-hospital mortality in a cohort of ICU patients with AECOPD, where potential confounding factors were mitigated through PSM. In this study of ICU patients with AECOPD, BUN levels were significantly higher in non-survivors compared to the survival group before and after PSM. Prior to PSM, multivariate analysis indicated that elevated BUN levels were independently associated with an increased risk of in-hospital mortality after adjusting for possible confounders. After PSM, high BUN levels were still associated with an increased risk of in-hospital mortality. Moreover, our findings show that BUN is an independent risk factor for patients with AECOPD. In the present study, before PSM, the optimal BUN cut-off value was 23 mg/dL. The hazard ratio (HR) for the high BUN group was 1.8987 compared to the low BUN group, indicating that the risk of in-hospital mortality in the high BUN group was 1.8987 times higher than in the low BUN group. Based on the Youden index, the optimal BUN cut-off value for in-hospital mortality in critical patients with AECOPD after PSM was 22 mg/dL, which was almost the same as before PSM. AECOPD patients with a high BUN group had an HR of 1.7358 compared with the low BUN group, implying that the high BUN group patients had a 1.7358 times higher risk of in-hospital death than the low BUN group.

Blood urea nitrogen (BUN) is not only an indicator of renal function but is also an effective marker of neurohormonal activity. BUN has been shown to be an important predictor of in-hospital mortality in various diseases [11,12,13]. In a retrospective study that included 4176 patients admitted to a German ICU, Arihan et al. [20] revealed that a high BUN level at admission was significantly associated with adverse outcomes in critically ill patients admitted to an ICU; this association existed even after correction for relevant confounders in multivariate analyses. Furthermore, previous studies [21,22] reported that the BUN/albumin ratio is an independent predictor of mortality in community-acquired and hospital-acquired pneumonia. Moreover, Küçükceran et al. [23] evaluated the association between serum BUN and albumin levels on admission in patients with COVID-19 in-hospital mortality. They demonstrated that BUN levels in the death group were considerably higher than in the survivor group, with an AUC of 0.771 for predicting in-hospital mortality. In the present study, we also found that a higher BUN level was associated with in-hospital mortality in AECOPD, with an AUC of 0.629 before PSM and 0.591 after PSM.

A recent study [24] found that high BUN levels at admission were significantly associated with hospital mortality in AECOPD patients who presented to the emergency department, and the optimal cut-off value for hospital mortality was 7.63 mmol/L. In this large, hospital-based propensity score matching study, we also found that AECOPD patients admitted to the ICU with higher baseline BUN levels (>23 mg/dL before PSM; >22 mg/dL after PSM) had a higher risk of mortality than those with lower BUN. Although the exact mechanism underlying this relationship is unclear, several mechanisms may be attributable to elevated BUN levels in AECOPD patients. First, exacerbation of COPD results in prolonged hypoxemia and hypercapnia, which activates the renin–angiotensin–aldosterone system (RAAS) and increases flow-dependent urea reabsorption in the distal tubules, resulting in increased BUN levels [9,25,26]. Secondly, cardiovascular comorbidities such as heart failure are common in COPD patients. In patients with cardiovascular disease, a complex neurohormonal mechanism is activated, which activates the renal sympathetic nervous system and the RAAS, resulting in urea reabsorption dysregulation [27]. Finally, COPD is linked to persistent blood and airway neutrophilia, as well as systemic and tissue hypoxia. Patients with COPD have higher levels of hypoxia-upregulated neutrophil-derived proteins and elevated concentrations of circulating proinflammatory mediators, which may affect the cardiorenal function and neurohumoral regulation, raising BUN levels [9,25,28].

Our results showed that the in-hospital mortality rate was 13.7% in AECOPD patients admitted to the ICU. Mohan et al. [29] also reported an in-hospital mortality rate of 13.7% in patients with AECOPD requiring admission to the ICU, which is consistent with our findings. In contrast, Warwick et al. [30] reported a high hospital mortality rate (27.2%) in patients with AECOPD admitted to the ICU. Variability in published mortality rates for AECOPD patients could be attributed to differences in patient characteristics, quality of care, prolonged ICU stay, and mechanical ventilation use. Furthermore, our findings imply that the risk of in-hospital mortality was found to be higher in critically ill patients with a BUN level >23 mg/dl. Our results suggest that BUN is a widely and rapidly available laboratory test that may allow physicians and hospital staff to identify COPD patients with increased risk of adverse outcomes requiring early intensive care.

This study has several limitations. First, this retrospective cohort study may have led to selection bias. Nevertheless, PSM and multivariate analyses were used to minimize selection bias and adjust for confounding variables. Second, although we used PSM to reduce bias, there may have been some residual and unmeasured confounders. Third, some individuals with cardiovascular comorbidities may have taken medications that influence BUN levels. However, even after adjusting for multiple comorbidities, BUN levels remained an independent predictor of in-hospital mortality. Fourth, the BUN levels could have been affected by many factors, such as diuretic use and protein intake; nevertheless, due to the study’s retrospective nature, these conditions could not be differentiated. Fifth, the AUC for BUN did not perform well in ROC analysis, which may have influenced the accuracy of the results. Finally, this is a single-center study, which limits the generalizability of our findings.

## 5. Conclusions

In this cohort study, elevated BUN levels were independently associated with increased in-hospital mortality in critically ill patients with AECOPD. BUN is a routine laboratory test that may be useful in the risk stratification of these patients. Future prospective multicenter studies with a larger sample size are needed to evaluate further the association between BUN level and mortality in patients with AECOPD.

## Figures and Tables

**Table 1:** Demographic and clinical characteristics of the study population.

	Before PSM			After PSM		
Characteristics	Survival (*n* = 1037)	Death (*n* = 164)	*p*	Survival (*n* = 159)	Death (*n* = 159)	*p* Value
* Age, year	71 (64–80)	76 (70–82.5)	0.001	74 (66–84)	76 (70–82.5)	0.258
* Gender, male	524 (50.5)	79 (48.2)	0.574	84 (52.8)	79 (49.7)	0.575
LOS hospital, day	8 (4.9–13.9)	8.5 (4.0–15.8)	0.666	8.8 (5.9–16.3)	8.7 (4–15.8)	0.227
LOS ICU, day	2.4 (1.3–4.9)	3.9 (1.7–7.9)	0.001	2.3 (1.2–4.6)	3.9 (1.7–7.9)	0.001
Laboratory tests						
* Hemoglobin, g/dL	11.2 (9.8–12.7)	10.9 (9.2–12.2)	0.001	10.4 (9.4–12.5)	10.9 (9.1–12.2)	0.797
* WBC, 10^9^/L	10.9 (7.9–14.6)	12.7 (9.2–17.5)	0.854	11.4 (8.1–15.6)	12.7 (9.3–17.2)	0.076
* Platelets, 10^9^/L	217 (163–280)	193 (133.2–271.7)	0.001	217 (149.8–265.5)	192 (133–276.5)	0.187
Glucose, mg/dL	144 (119.4–180.3)	144 (121.7–178.4)	0.828	138.6 (117.5–175)	142.3 (118.8–174)	0.501
* Creatinine, ng/dL	1 (0.8–1.5)	1.1 (0.8–1.8)	0.040	1.1 (0.8–1.6)	1.1 (0.8–1.8)	0.618
MCHC, g/dL	32.2 (31.1–33.2)	32.1 (31–33.2)	0.377	31.9 (30.9–33)	32.1 (31–33.2)	0.456
* RDW, %	14.8 (13.8–16.1)	15.2 (14–16.9)	0.006	15.1 (14.2–17)	15.2 (14–16.9)	0.958
* Hematocrit, %	35 (30.5–39.5)	33.7 (28.9–37.7)	0.004	33.7 (29.1–38)	33.4 (28.2–37.6)	0.698
BUN, mg/dL	22.5 (16–35.5)	30 (20.5–45)	0.001	25.5 (16.5–37.5)	30 (20.5–44.5)	0.005
* Bicarbonate, mEq/L	26 (23–30)	25 (21.3–30)	0.036	26 (22.5–29)	25 (21.5–30)	0.269
Comorbidities						
* Hypertension	423 (40.8)	57 (34.8)	0.146	57 (35.8)	55 (34.6)	0.907
* Diabetes	321 (31)	48 (29.3)	0.716	47 (29.6)	46 (28.9)	0.902
* Congestive heart failure	516 (49.8)	93 (56.7)	0.110	87 (54.7)	90 (56.6)	0.735
* Coronary artery disease	309 (29.8)	57 (34.8)	0.202	45 (28.5)	55 (34.6)	0.227
* Chronic kidney disease	247 (23.8)	41 (25)	0.768	39 (25.5)	40 (25.2)	0.897
* Severe liver disease	18 (1.7)	5 (3)	0.228	4 (2.5)	5 (3.1)	0.735
* Obesity	161 (15.5)	24 (14.5)	0.817	25 (17.5)	24 (15.1)	0.877
* Malignant cancer	132 (12.7)	44 (26.8)	0.001	37 (23.3)	41 (25.8)	0.602
* Cerebrovascular disease	82 (7.9)	17 (10.4)	0.286	15 (9.4)	17 (10.7)	0.709
Monitoring parameters						
* Heart rate, Bpm	87 (77–99)	91 (79–106)	0.004	89 (80–102)	91 (79–105)	0.654
* MAP, mmHg	78 (71–85)	75 (69–82)	0.004	77 (71–84)	75 (69–82)	0.101
* RR, breaths/minutes	20 (18.1–23)	21 (19–24)	0.022	21 (19–24)	21 (19–24)	0.588
SpO2,%	95.4 (93.8–97)	95 (93.5–97.3)	0.649	95 (94–97)	95 (93–97)	0.834
Intervention						
* Mechanical ventilation	327 (31.5)	66 (40.2)	0.031	50 (31.4)	62 (39)	0.159

Values are expressed as the median (IQR) or n (%). PSM, propensity score matching; LOS, length of stay; ICU, intensive care unit; WBC, white blood cells; MCHC, mean corpuscular hemoglobin concentration; RDW, red blood cell distribution width; BUN, blood urea nitrogen; MAP, mean arterial pressure; RR, respiratory rate. * Covariables included in the PSM.

**Table 2:** Binomial logistic regression analysis of BUN for in-hospital mortality among critically ill patients with AECOPD (before PSM).

Variable	Univariate Analysis		Multivariate Analysis	
	OR (95% CI)	*p*	OR (95% CI)	*p*
Age	1.039 (1.022–1.056)	0.001	1.042 (1.023–1.061)	0.001
Gender (male)	0.910 (0.574–0.910)	0.574		
LOS hospital	1.005 (0.987–1.023)	0.606		
LOS ICU	1.047 (1.021–1.073)	0.001	1.038 (1.008–1.069)	0.012
Hemoglobin	0.897 (0.829–0.970)	0.006	1.078 (0.758–1.532)	0.675
WBC	1.040 (1.017–1.063)	0.001	1.029 (1.004–1.054)	0.021
Platelets	0.998 (0.997–1.000)	0.049	0.998 (0.996–1.000)	0.033
Glucose	1.002 (0.999–1.005)	0.206		
Creatinine	1.087 (0.949–1.243)	0.228		
MCHC	0.958 (0.865–1.060)	0.401		
RDW	1.113 (1.042–1.188)	0.002	1.076 (0.988–1.172)	0.093
Hematocrit	0.966 (0.942–0.992)	0.010	0.974 (0.872–1.089)	0.644
BUN	1.018 (1.010–1.025)	0.001	1.014 (1.005–1.022)	0.002
Bicarbonate	0.972 (0.944–1.001)	0.059	1.012 (0.979–1.046)	0.485
Hypertension (yes)	0.773 (0.548–1.091)	0.143		
Diabetes (yes)	0.923 (0.643–1.325)	0.664		
Congestive heart failure (yes)	1.323 (0.949–1.843)	0.099	1.108 (0.762–1.609)	0.592
Coronary artery disease (yes)	1.255 (0.886–1.777)	0.201		
Chronic kidney disease (yes)	1.066 (0.728–1.561)	0.742		
Severe liver disease (yes)	1.780 (0.652–4.862)	0.261		
Obesity (yes)	0.933 (0.586–1.484)	0.769		
Malignant cancer (yes)	2.514 (1.701–3.716)	0.001	2.971 (1.929–4.577)	0.001
Cerebrovascular disease (yes)	1.347 (0.777–2.335)	0.289		
Hear rate	1.016 (1.005–1.027)	0.003	1.018 (1.005–1.030)	0.005
Mean arterial pressure	0.975 (0.959–0.992)	0.015	0.993 (0.974–1.012)	0.447
Respiratory rate	1.056 (1.011–1.104)	0.001	1.030 (0.979–1.084)	0.259
SpO2	0.966 (0.900–1.036)	0.332		
Mechanical ventilation (yes)	1.462 (1.042–2.051)	0.028	1.654 (1.093–2.505)	0.017

AECOPD, acute exacerbation of chronic obstructive pulmonary disease; PSM, propensity score matching; OR, odds ratio; CI, confidence interval; LOS, length of stay; ICU, intensive care unit; WBC, white blood cells; BUN, blood urea nitrogen; MCHC, mean corpuscular hemoglobin concentration; RDW, red blood cell distribution width.

**Table 3:** Binomial logistic regression analysis of BUN for in-hospital mortality among critically ill patients with AECOPD (after PSM).

Variable	Univariate Analysis		Multivariate Analysis	
	OR (95% CI)	*p*	OR (95% CI)	*p*
Age	1.013 (0.992–1.035)	0.233		
Gender (male)	0.882 (0.568–1.369)	0.575		
LOS hospital	0.999 (0.976–1.022)	0.919		
LOS ICU	1.075 (1.026–1.172)	0.002	1.077 (1.027–1.130)	0.002
Hemoglobin	0.993 (0.901–1.095)	0.890		
WBC	1.008 (0.980–1.036)	0.571		
Platelets	0.999 (0.997–1.001)	0.575		
Glucose	1.022 (0.998–1.005)	0.375		
Creatinine	1.017 (0.806–1.283)	0.885		
MCHC	1.048 (0.920–1.193)	0.481		
RDW	1.009 (0.927–1.099)	0.830		
Hematocrit	0.995 (0.964–1.027)	0.747		
BUN	1.016 (1.004–1.027)	0.008	1.015 (1.003–1.027)	0.015
Bicarbonate	0.984 (0.948–1.022)	0.408		
Hypertension (yes)	0.946 (0.597–1.499)	0.814		
Diabetes (yes)	0.970 (0.598–1.573)	0.902		
Congestive heart failure (yes)	1.079 (0.693–1.680)	0.735		
Coronary artery disease (yes)	1.340 (0.833–2.155)	0.228		
Chronic kidney disease (yes)	1.034 (0.622–1.720)	0.897		
Severe liver disease (yes)	1.258 (0.332–4.774)	0.736		
Obesity (yes)	0.953 (0.518–1.752)	0.877		
Malignant cancer (yes)	1.146 (0.687–1.911)	0.602		
Cerebrovascular disease (yes)	1.149 (0.553–2.390)	0.709		
Heart rate	1.002 (0.988–1.016)	0.815		
Mean arterial pressure	0.981 (0.960–1.002)	0.081	0.987 (0.965–1.009)	0.251
Respiratory rate	1.016 (0.962–1.074)	0.566		
SpO2	0.967 (0.886–1.057)	0.464		
Mechanical ventilation (yes)	1.393 (0.878–2.212)	0.159		

AECOPD, acute exacerbation of chronic obstructive pulmonary disease; PSM, propensity score matching; OR, odds ratio; CI, confidence interval; LOS, length of stay; ICU, intensive care unit; WBC, white blood cells; BUN, blood urea nitrogen; MCHC, mean corpuscular hemoglobin concentration; RDW, red blood cell distribution width.

## Data Availability

The raw data used in this study were available from MIMIC-IV, a freely accessible critical care database (https://mimic.physionet.org/gettingstarted/access) (accessed on 28 September 2021). We completed the online course and passed the online exams to access and use the data (record ID: 45355193).
